# 25(OH)D Is Effective to Repress Human Cholangiocarcinoma Cell Growth through the Conversion of 25(OH)D to 1α,25(OH)_2_D_3_

**DOI:** 10.3390/ijms17081326

**Published:** 2016-08-12

**Authors:** Kun-Chun Chiang, Chun-Nan Yeh, Cheng-Cheng Huang, Ta-Sen Yeh, Jong-Hwei S. Pang, Jun-Te Hsu, Li-Wei Chen, Sheng-Fong Kuo, Atsushi Kittaka, Tai C. Chen, Horng-Heng Juang

**Affiliations:** 1 General Surgery Department and Zebrafish Center, Chang Gung Memorial Hospital, Chang Gung University, Keelung 204, Taiwan; 2 General Surgery Department, Chang Gung Memorial Hospital, Chang Gung University, Kwei-Shan, Taoyuan 244, Taiwan; yehchunnan@gmail.com (C.-N.Y.); tsy471027@adm.cgmh.org.tw (T.-S.Y.); hsujt2813@adm.cgmh.org.tw (J.-T.H.); 3 Pathology Department, Chang Gung Memorial Hospital, Chang Gung University, Keelung 204, Taiwan; rrooyyaall@yahoo.com; 4 Graduate Institute of Clinical Medical Sciences, College of Medicine, Chang Gung University, Kwei-Shan, Taoyuan 244, Taiwan; jonghwei@mail.cgu.edu.tw; 5 Department of Gastroenterology, Chang Gung Memorial Hospital, Chang Gung University, Keelung 204, Taiwan; leiwei@adm.cgmh.org.tw; 6 Department of Endocrinology and Metabolism, Chang Gung Memorial Hospital, Chang Gung University, Keelung 204, Taiwan; shengfoung@gmail.com; 7 Faculty of Pharmaceutical Sciences, Teikyo University, 2-11-1 Kaga, Itabashi, Tokyo 173-8605, Japan; akittaka@pharm.teikyo-u.ac.jp; 8 Endocrine core lab, boston University School of Medicine, Boston, MA 02118, USA; taichen@bu.edu; 9 Department of Anatomy, College of Medicine, Chang Gung University, Kwei-Shan, Taoyuan 244, Taiwan

**Keywords:** cholangiocarcinoma, 25(OH)D, CYP27B1, 1α-OHase, vitamin D

## Abstract

Cholangiocarcinoma (CCA) is a devastating disease without effective treatments. 1α,25(OH)_2_D_3_, the active form of Vitamin D, has emerged as a new anti-cancer regimen. However, the side effect of hypercalcemia impedes its systemic administration. 25(OH)D is biologically inert and needs hydroxylation by CYP27B1 to form 1α,25(OH)_2_D_3_, which is originally believed to only take place in kidneys. Recently, the extra-renal expression of CYP27B1 has been identified and in vitro conversion of 25(OH)D to 1α,25(OH)_2_D_3_ has been found in some cancer cells with CYP27B1 expression. In this study, CYP27B1 expression was demonstrated in CCA cells and human CCA specimens. 25(OH)D effectively represses SNU308 cells growth, which was strengthened or attenuated as CYP27B1 overexpression or knockdown. Lipocalcin-2 (LCN2) was also found to be repressed by 25(OH)D. After treatment with 800 ng/mL 25(OH)D, the intracellular 1α,25(OH)_2_D_3_ concentration was higher in SNU308 cells with CYP27B1 overexpression than wild type SNU308 cells. In a xenograft animal experiment, 25(OH)D, at a dose of 6 μg/kg or 20 μg/kg, significantly inhibited SNU308 cells’ growth without inducing obvious side effects. Collectively, our results indicated that SNU308 cells were able to convert 25(OH)D to 1α,25(OH)_2_D_3_ and 25(OH)D CYP27B1 gene therapy could be deemed as a promising therapeutic direction for CCA.

## 1. Introduction

Cholangiocarcinoma (CCA), the second most primary liver malignancy, accounts for 10%–15% of primary liver cancers with an increase of incidence and mortality recently [1,2]. Radical surgery is the most effective treatment for CCA, however, the late diagnosis and high recurrent rate usually render CCA patients unfit to receive operation [3]. Adding that traditional chemotherapy and radiotherapy fail to improve CCA patients’ survival, finding new therapeutic directions and regimens for CCA should be prioritized.

Vitamin D is originally deemed to only have mineral functions, but has become more and more popular in recent decades due to the unveiling of its non-mineral functions, such as pro-differentiation, pro-apoptosis, anti-angiogenesis, and anti-growth [4]. Thus, 1α,25(OH)_2_D_3_, the active form of Vitamin D, has emerged as a new regimen for cancer treatment [5]. To overcome the drawback of hypercalcemia induced by systemic administration of 1α,25(OH)_2_D_3_, thousands of 1α,25(OH)_2_D_3_ analogs have been synthesized aiming to lessen the hypercalcemia-inducing effect and to strengthen anti-tumor effect [6]. Besides Vitamin D analogs, another way to apply Vitamin D in cancer treatment is the usage of 25(OH)D.

After obtaining Vitamin D through intake or photo-conversion in the skin, Vitamin D is bound with Vitamin D binding protein (VDP) and then carried to the liver to be hydroxylated to form 25-hydroxyvitamin D [25(OH)D] [7], which is also the best index of human Vitamin D status and biological inert. 25(OH)D would be further hydroxylated to form 1α,25(OH)_2_D_3_ in the kidney, which is catalyzed by 25(OH)D-1α-hydroxylase (1α-OHase or CYP27B1). The finding that CYP27B1 exists in a variety of human tissues in addition to the kidney only [8,9,10] implies the extra-renal conversion of 25(OH)D to 1α,25(OH)_2_D_3_ seems reasonable. In fact, the extra-renal conversion had been proven, and the converted 1α,25(OH)_2_D works in an intra-, auto-, or para- crine manner [11,12], which indicates the application of 25(OH)D as a systemic treatment is a safe and feasible way to provide tissues with active Vitamin D. Previously, the conversion of 25(OH)D to 1α,25(OH)_2_D_3_ had been demonstrated in prostate and pancreas cancer cells with 25(OH)D exhibiting significant growth inhibition against cancer cells [13,14]. Our group also showed 25(OH)D could be converted to 1α,25(OH)_2_D_3_ by hepatocellular carcinoma cells and could repress hepatocellular carcinoma cell growth [15].

In this work, we aimed to show the conversion of 25(OH)D to 1α,25(OH)_2_D_3_ by CCA cells and application of 25(OH)D to treat CCA cells in vitro and in vivo. Through our work, we hope we can provide a new therapeutic regimen for CCA treatment.

## 2. Result

### 2.1. SNU308 Cells Expressed CYP27B1 Expressions and 25(OH)D Inhibited SNU308 Cells Growth

**Figure 1:**
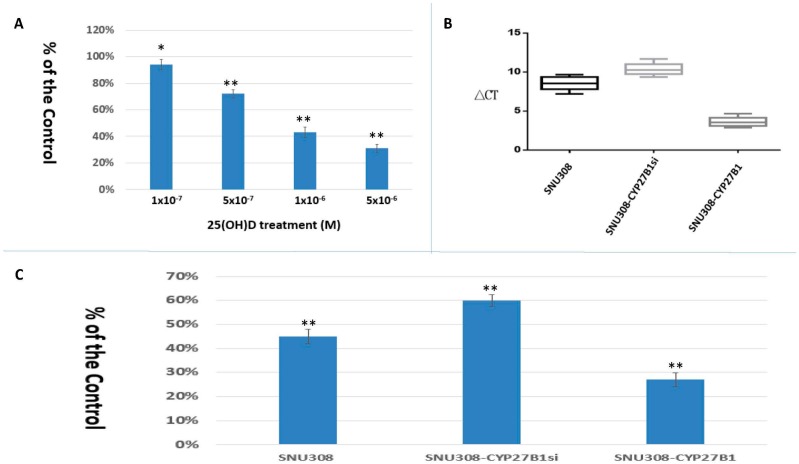
Anti-proliferative effects of 25(OH)D on SNU308, SNU308-CYP27B1, and SNU308-CYP27B1si cells (**A**) Two, four, and six days after plating, cells were treated by indicated concentrations of 25(OH)D and cell proliferation was measured by WST-1 method; (**B**) CYP27B1 mRNA expression of SNU308, SNU308-CYP27B1si, and SNU308-CYP27B1 cells; (**C**) The cell proliferation of SNU308, SNU308-CYP27B1si, and SNU308-CYP27B1 cells after 10^−6^ M 25(OH)D treatment was measured by WST-1 method. Each value is a mean ± SD of three to five determinations. * *p* < 0.05, ** *p* < 0.001 versus control.

The effect of 25(OH)D on SNU308 cell growth was evaluated by CyQUANT proliferation assay kit. As shown in Figure 1A, 1 × 10^−7^, 5 × 10^−7^, 1 × 10^−6^, and 5 × 10^−6^ M 25(OH)D treatments (7 days) suppressed SNU308 cell growth to 94% ± 4%, 72% ± 3%, 43% ± 3%, and 31% ± 3% of the control, respectively. We then transfected CYP27B1 into SNU308 cells (SNU308-CYP27B1) and knocked down CYP27B1in SNU308 cells (SNU308-CYP27B1si). Figure 1B shows that ∆CT of SNU308, SNU308-CYP27B1si, and SNU308-CYP27B1 cells was 8.24, 10.21, and 3.32, respectively. As treated by 1 × 10^−6^ M 25(OH)D for 7 days, 55% ± 3%, 40% ± 2.5%, and 73% ± 2.9% growth inhibition was observed in SNU308, SNU308-CYP27B1, and SNU308-CYP27B1si cells, respectively (Figure 1C). Our result indicates that SNU308 cells were growth-inhibited by 25(OH)D and overexpression or knockdown of CYP27B1 could strengthen or attenuate the 25(OH)D-induced growth inhibition. A similar inhibitory effect of 25(OH)D on SNU1079 cells was also observed (Supplementary Material Figure S1).

### 2.2. Evaluation of 25(OH)D Effect on LCN2 Expression in SNU308 Cells

**Figure 2:**
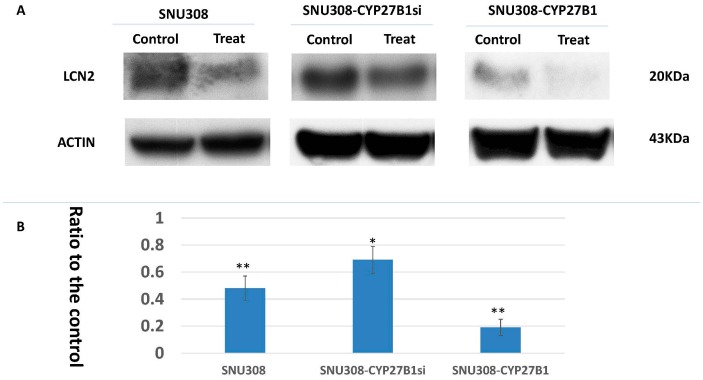
Evaluation of 25(OH)D effect on LCN2 expression in SNU308 cells with or without modulation of CYP27B1 gene expression. (**A**) A Western blot depicting LCN2 expression in SNU308, SNU308-CYP27B1si, and SNU308-CYP27B1 cells after two days of 10^−6^ M 25(OH)D treatment; (**B**) Quantitative analysis of LCN2 expression. Each value is a mean ± SD of three to five determinations. * *p* < 0.05, ** *p* < 0.001 versus control.

LCN2 had been shown to be one of 1α,25(OH)_2_D_3_ downstream genes in SNU308 cells [16]. In this current study, we thus investigated LCN2 expression in SNU308 cells after 25(OH)D treatment. As shown in Figure 2, LCN2 expression was repressed by 10^−6^ M 25(OH)D in SNU308, SNU308-CYP27B1si, and SNU308-CYP27B1 cells to 0.48 ± 0.09, 0.69 ± 0.1, and 0.19 ± 0.06-fold, respectively.

### 2.3. Evaluation of Conversion of 25(OH)D to 1α,25(OH)_2_D_3_ in SNU308 Cells

**Figure 3:**
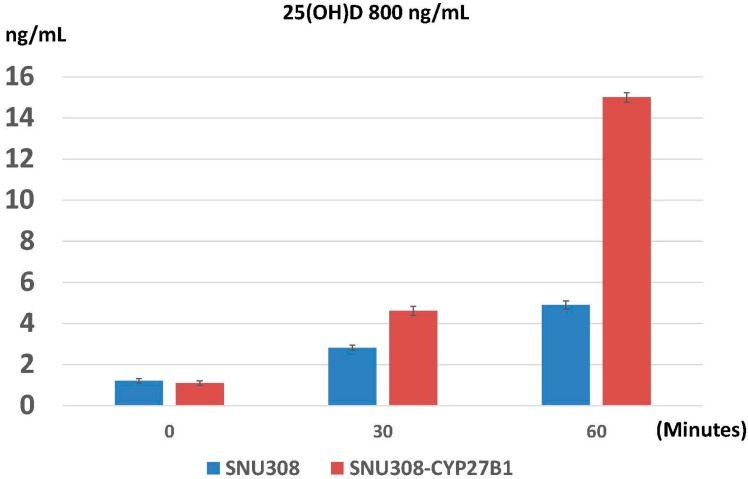
Evaluation of intracellular 1α,25(OH)_2_D_3_ concentration after 25(OH)D treatment in SNU308 and SNU308-CYP27B1 cells. The intracellular 1α,25(OH)_2_D_3_ concentrations of SNU308 and SNU308-CYP27B1 cells after 800 ng/mL 25(OH)D treatment were measured by ELISA at indicated time points. Each value is a mean ± SD of three to five determinations.

Since 25(OH)D is biologically inactive, the growth inhibition effect of 25(OH)D on SNU308 cells needs conversion of 25(OH)D to 1α,25(OH)_2_D_3_. To investigate whether the conversion exists or not, SNU308 and SNU308-CYP27B1 cells were treated with 800 ng/mL 25(OH)D and intracellular 1α,25(OH)_2_D_3_ concentration was determined by ELISA. As shown in Figure 3, 2.8 and 4.9 ng/mL of 1α,25(OH)_2_D_3_ were found in SNU308 cells at 30 and 60 min after treatment; while 4.6 and 15 ng/mL of 1α,25(OH)_2_D_3_ were noted in SNU308-CYP27B1 cells. Our result indicates that SNU308 cells were capable of converting 25(OH)D to 1α,25(OH)_2_D_3_, leading to the growth inhibition induced by 25(OH)D.

### 2.4. Evaluation the Anti-Growth Effect of 25(OH)D on SNU308 Cells in Vivo

**Figure 4:**
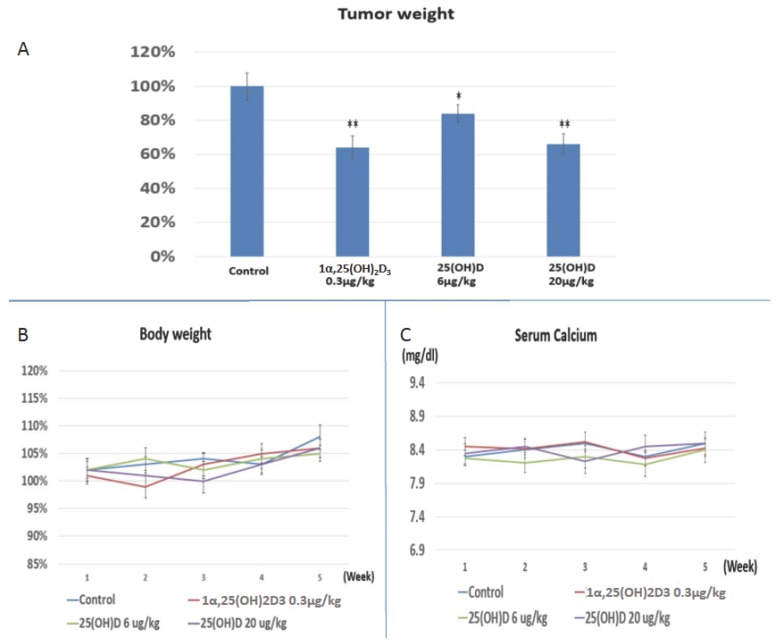
Evaluation of 25(OH)D and 1α,25(OH)_2_D_3_ effect on SNU308 cells growth in vivo through a xenograft animal model. (**A**) Four weeks after treatment, the xenografted tumors from each group were weighed; (**B**) The body weight of nude mice in each group; (**C**) The serum calcium concentration of nude mice in each group. Each value is a mean ± SD of six determinations. * *p* < 0.05, ** *p* < 0.001 versus control.

Since SNU308 cells possessed the ability to convert 25(OH)D to 1α,25(OH)_2_D_3_, we thus applied 25(OH)D to treat xenografted SNU308 cells in nude mice. Figure 4A shows that 1α,25(OH)_2_D_3_ treatment (0.3 μg/kg, twice per week) reduced tumor weight to 64% ± 7% of the control; while 25(OH)D treatment reduced tumor weight to 84% ± 5% or 66% ± 6% of the control, at the dose of 6 μg/kg or 20 μg/kg, twice per week. The mice body weight and serum calcium did not change as obviously in the treated groups compared to the control group (Figure 4B,C). Our result indicates that both 25(OH)D and 1α,25(OH)_2_D_3_ could repress SNU308 cell growth in vivo, suggesting the in vivo conversion of 25(OH)D to 1α,25(OH)_2_D_3_ by SNU308 cells.

### 2.5. Evaluation of CYP27B1 Expression in Human CCA Specimens

**Figure 5:**
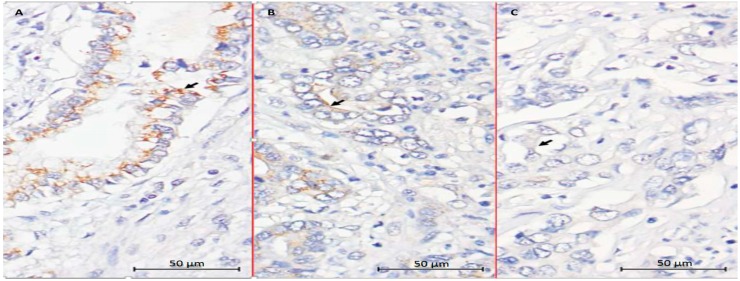
CYP27B1 expression in human CCA. Immunohistochemical staining of CYP27B1 in human CCA specimens with different intensity scores. (**A**) High expression; (**B**) Moderate expression; (**C**) Low expression; (×400, Scale bar = 50 µm). ( Arrows indicate positive expression of CYP27B1).

We next analyzed CYP 27B1 expression in 83 CCA patients undergoing hepatectomy by immunohistochemical staining. The result of IHC showed that 33 (39.8%), 36 (43.4%), and 14 (16.9%) specimens were categorized as having weak, moderate, and strong staining intensity for CYP27B1, respectively (Figure 5).

## 3. Discussion

In this study, we demonstrated that SNU308 cells presented with CYP27B1 expression and were growth inhibited by 25(OH)D in a dose dependent manner. The inhibitor effect of 25(OH)D on SNU308 cells growth was strengthened or weakened as CYP27B1 overexpression or knockdown. A similar effect of 25(OH)D on SNU308 cells’ LCN2 expression was observed. The above findings all suggested that SNU308 cells were able to convert 25(OH)D to 1α,25(OH)_2_D_3_, leading to the inhibition of SNU308 cells’ growth and LCN2 expression. As we measured intracellular 1α,25(OH)_2_D_3_ concentration of SNU308 cells after 25(OH)D treatment, SNU308-CYP27B1 cells had higher 1α,25(OH)_2_D_3_ concentration than SNU308 cells, confirming the conversion of 25(OH)D to 1α,25(OH)_2_D_3_ by SNU308 cells. 1α,25(OH)_2_D_3_ (0.3 μg/kg) and 25(OH)D (6 and 20 μg/kg) were then applied to treat SNU308 cells in a xenograft model and a significant anti-tumor growth effect was induced by both 25(OH)D and 1α,25(OH)_2_D_3_ with no obvious side effects noted in both groups. We further showed that CYP27B1 expression in human CCA specimens. Collectively, our result indicated that 25(OH)D was a promising and safe regimen for CCA treatment.

CYP27B1—an enzyme that functions to hydroxylate 25(OH)D to form 1α,25(OH)_2_D_3_ and the active form of Vitamin D—was generally considered to only be expressed in the kidney under normal physical conditions until the mid-1980s. Extra-renal CYP27B1 expression was found during the ensuing years, with Bike at al. reporting the first extra-renal synthesis of 1α,25(OH)_2_D_3_ in cultured human keratinocytes [17]. So far, a variety of cells have been reported to have CYP27B1 expression and could convert 25(OH)D to 1α,25(OH)_2_D_3_, such as prostate, breast, pancreas, and liver [14,15,18,19]. Adding that the extra-renal converted 1α,25(OH)_2_D_3_ works in a para- or endo-crine manner which suggests the safety of systemic application of 25(OH)D. 25(OH)D has therefore emerged as a promising regimen to treat cancers with CYP27B1 expression. This concept is supported by the epidemiological studies showing Vitamin D deficiency is associated with higher incidence of cancers [20,21,22,23], for which the explanation was Vitamin D deficiency represented lower circulated 25(OH)D concentration, leading to the fewer converted 1α,25(OH)_2_D_3_ in the extra-renal tissues due to the fewer substrates for CYP27B1 to convert. Our data clearly showed that 25(OH)D could effectively repress growth of SNU308 cells in vitro (Figure 1A). As we xenografted SNU308 cells into nude mice and treated them with 25(OH)D and 1α,25(OH)_2_D_3_ through intraperitoneal injection, both drugs exhibited potent anti-growth effect on xenografted tumors (Figure 4A). These results suggested that SNU308 cells could convert 25(OH)D to 1α,25(OH)_2_D_3_ both in vitro and in vivo, resulting in the growth inhibition of 25(OH)D against SNU308 cells. The steadily increased body weight and constant serum calcium concentration of nude mice during the experimental period indicated the safety of systemic application of 25(OH)D (Figure 4B,C). Collectively, our results indicated that 25(OH)D constituted an effective and safe regimen to treat CCA.

To further confirm the conversion of 25(OH)D to 1α,25(OH)_2_D_3_ by SNU308 cells, CYP27B1 was then overexpressed or knocked down in SNU308 cells. Figure 1C shows that the growth inhibition effect of 25(OH)D on SNU308 cells was increased or decreased as CYP27B1 forced expression or knock down, respectively, which suggested that SNU308 cells could convert 25(OH)D to 1α,25(OH)_2_D_3_. The results shown in Figure 3 further confirmed the conversion since SNU308-CYP27B1 cells could yield higher concentration of 1α,25(OH)_2_D_3_ than SNU308 cells as treated by 25(OH)D, which was measured by ELISA. The finding that the transfection of CYP27B1 into SNU308 cells could strengthen the anti-growth effect of 25(OH)D also implicated the possibility of CYP27B1 gene transfection therapy for CCA (Figure 1C). In fact, CYP27B1 gene expression had been negatively correlated with invasiveness of ovarian cancer [24]. In this current study, we demonstrated that human CCA specimen presented with CYP27B1 expression (Figure 5), which encouraged the application of 25(OH)D in clinical trials for CCA treatment.

1α,25(OH)_2_D_3_ modulates the downstream gene expressions through binding to Vitamin D receptor (VDR), which would further conjugate with retinoid X receptor (RXR) to form a heterodimer. The heterodimer then binds to Vitamin D response element (VDRE) to modulate gene expression [25,26]. Lipocalin-2 (LCN2), belonging to the lipocalin superfamily, was originally deemed as a stress protein [27], with the oncogene role of LCN2 later being explored in a variety of cancers [16,28,29,30], including CCA. Previously, we had demonstrated that 1α,25(OH)_2_D_3_ could inhibit LCN2 expression in CCA cells and knockdown of VDR could abolish this effect, indicating LCN2 was susceptible to 1α,25(OH)_2_D_3_ in a VDR dependent manner [16]. Since 25(OH)D is inert biologically, the downregulation of LCN2 in SNU308 cells as treated by 25(OH)D suggested the added 25(OH)D was converted to 1α,25(OH)_2_D_3_, leading to the inhibition of LCN2 (Figure 2). The increase or decrease of LCN2 inhibition noted in SNU308-CYP27B1 or SNU308-CYP27B1si cells further confirmed the extra-renal synthesis of 1α,25(OH)_2_D_3_ by SNU308 cells.

## 4. Materials and Methods

### 4.1. Cell Culture and Chemicals

1α,25-dihydroxyvitamin D and 25-dihydroxyvitamin D were obtained from Sigma-Aldrich Co. (St. Louis, MO, USA). SNU 308 cells, human CCA cell lines, was purchased from Korean Cell Line Bank (KCLB: 28 Yongon-dong, Chongno-gu, Seoul, Korea). Cells were grown in RPMI 1640 medium supplemented with 10% FBS and 1% antibiotic-antimycotic agents. Culture medium was changed 3 times per week.

### 4.2. Western Blot Assay

The detailed procedures for Western blot were described previously [31]. The primary antibodies used in this study was monoclonal antibodies against LCN2 (#PAB9543, Abnova Corporation, Taipei, Taiwan). The secondary antibodies (1:5000) were anti-rabbit (111-035-003, Jackson Immunoresearch, West Grove, PA, USA) or anti-mouse secondary antibodies (Zymed 81-6520, San Francisco, CA, USA). The blots were detected using ECL reagents (WBKLS0500, Millipore, Billerica, MA, USA). Membranes were detected by VersaDoc^TM^ Imaging System (Bio-Rad, Hercules, CA, USA) for analysis.

### 4.3. Cell Proliferation Assay

The cell proliferation of SNU308 cells with or without treatment was determined using CyQUANT cell proliferation assay kit (Invitrogen, Carlsbad, CA, USA).

### 4.4. Knockdown CYP27B1

SNU308 cells were transduced with CYP27B1 small hairpin RNA lentiviral particles (sc-60479-V; Santa Cruz Biotechnology) as described by the manufacturer. Two days after transduction, the cells (SNU308-CYP27B1si) were selected with puromycin dihydrochloride.

### 4.5. Real-Time Reverse Transcription-Polymerase Chain Reaction (RT-qPCR)

Total RNA from cells was isolated using Trizol reagent, cDNA was synthesized, and real-time polymerase chain reaction (qPCR) was performed according to the manufacturer’s protocol. FAM dye-labeled TaqMan MGB probes and PCR primers for human CYP27B1 (Hs00168017_m1) was purchased from Applied Biosystems (Foster City, CA, USA). β-actin (Hs01060665_g1) was used with a FAM reporter dye-labeled TaqMan MGB probe as an internal control.

### 4.6. Expression Vector Constructs and Stable Transfection

The human CYP27B1 expression vectors were constructed by ligation the CYP27B1 cDNA (cat # SC123873, Origene, Rockville, MD, USA) into the pcDNA3.1/Zeo expression vector (Invitrogen) with *Eco R1* and *Xba 1* cutting site. Proper ligation was confirmed by extensive restriction mapping and sequencing. Electroporation was performed using the ECM 830 (BTX, San Diego, CA, USA) with a single 70 ms pulse of 180V, and transfected SNU308 (SNU308-CYP27B1) cells were selected in a RPMI medium with 10% FCS and 100 μg/mL Zeocin (Invitrogen) as described before [15].

### 4.7. Measurement of 1α,25(OH)_2_D_3_

The detailed procedures were accorded to the manufacturer’s protocol (#E01D0002, BLUE GENE). Each well was treated by 800 ng/mL 25(OH)D and the cells were collected at indicated time points. 1α,25(OH)_2_D_3_ concentration was measured by ELISA (#E01D0002, BLUE GENE).

### 4.8. Xenograft Animal Study

The study was approved by the Chang Gung University Animal Research Committee (Permit Number: 2014022601). Young male BALB/C Nu/Nu mice, age 6 weeks old and average weight 20–30 g, were purchased from National Taiwan Animal Center. A total of 24 adult male Young male BALB/C Nu/Nu mice were used in this experiment. 5 × 10^6^ of SNU-308 cells were resuspended in 100 μL of PBS and injected into the subcutaneous. One week after tumor injection, different treatments were started. Four groups were included in this study, i.e., ethanol treatment group as the shame group (twice per week, intraperitoneal injection, *n* = 6), 1α,25(OH)_2_D_3_ treatment group (0.3 μg/kg, twice per week, intraperitoneal injection, *n* = 6), 25(OH)D low-dose group (6 μg/kg, twice per week, intraperitoneal injection, *n* = 6), and 25(OH)D high-dose group (20 μg/kg, twice per week, intraperitoneal injection, *n* = 6). The body weight and blood calcium were measured weekly. Tumor masses were harvested after 5 weeks. Blood calcium was measured using quantitative colorimetric calcium assay kits (BioChain, Newark, CA, USA) according to the manufacturer’s protocol.

### 4.9. CYP 27B1 Immunohistochemistry

The study was approved by the local institutional review board of Chang Gung Memorial Hospital (clinical study numbers 99-2886B, 99-3810B and 102-5813B), and written informed consent for immunohistochemical tumor analysis was obtained from each patient. CYP27B1 expression levels in the 83 CCA patients were examined by immunohistochemical staining (IHC). Tissue sections (4-μm) prepared from the formalin-fixed, paraffin-embedded hepatectomy specimens were incubated with the primary antibody against CYP27B1 (AP9056b 1:100 dilution; Abgent, San Diego, CA, USA) at 4 °C overnight. After 3-time washes with TBST (5 min each), the signals were visualized with the Dako Labelled Streptavidin-Biotin2 (LSAB2) System-HRP (Dako A/S, No. K0675; Dako, Glostrup, Denmark). Control slides were incubated with the secondary antibody only. For the assessment of immunohistochemical staining, the percentage of stained target cells was evaluated in 10 random microscopic fields per tissue section (×400 magnification), and their averages were subsequently calculated. Staining intensities were scored as 0, (negative), 1 (negative to weak), 2 (weak), 3 (weak to moderate), 4 (moderate), 5 (moderate to strong), or 6 (strong). Specimens with staining intensity scores of ≤2, 3–4, or 5–6 were classified as having weak, moderate, or strong expression, respectively.

### 4.10. Statistical Analysis

The data from each group were compared by two sample, unpaired, and two tails *t-*test. For animal studies, Mann-Whitney U test was applied. *p-*Value < 0.05 was considered as a significant difference.

## 5. Conclusions

Our results demonstrated that CCA cells demonstrated CYP27B1 expression and were able to convert 25(OH)D to 1α,25(OH)_2_D_3_. 25(OH)D was an effective and safe agent to repress growth of SNU308 cells in vitro and in vivo. CYP27B1 gene transfection would strengthen 25(OH)D-induced growth inhibition in SNU308 cells. Adding the finding that human CCA specimens exhibited CYP27B1 expression, application of 25(OH)D and CYP27B1 gene transfection to treat CCA seem to be promising approaches. Further clinical trials are justified.

## Supplementary Materials

Supplementary materials can be found at www.mdpi.com/1422-0067/17/8/1326/s1.

## References

[1] Gores G.J. (2003). Cholangiocarcinoma: Current concepts and insights. Hepatology.

[2] Shaib Y., El-Serag H.B. (2004). The epidemiology of cholangiocarcinoma. Semin. Liver Dis..

[3] Wang Y., Li J., Xia Y., Gong R., Wang K., Yan Z., Wan X., Liu G., Wu D., Shi L. (2013). Prognostic nomogram for intrahepatic cholangiocarcinoma after partial hepatectomy. J. Clin. Oncol..

[4] Chiang K.C., Chen T.C. (2013). The anti-cancer actions of vitamin D. Anticancer Agents Med. Chem..

[5] Deeb K.K., Trump D.L., Johnson C.S. (2007). Vitamin D signalling pathways in cancer: Potential for anticancer therapeutics. Nat. Rev. Cancer.

[6] Leyssens C., Verlinden L., Verstuyf A. (2014). The future of vitamin D analogs. Front. Physiol..

[7] Schuster I. (2011). Cytochromes p450 are essential players in the vitamin d signaling system. Biochim. Biophys. Acta.

[8] Townsend K., Banwell C.M., Guy M., Colston K.W., Mansi J.L., Stewart P.M., Campbell M.J., Hewison M. (2005). Autocrine metabolism of vitamin D in normal and malignant breast tissue. Clin. Cancer Res..

[9] Zehnder D., Bland R., Williams M.C., McNinch R.W., Howie A.J., Stewart P.M., Hewison M. (2001). Extrarenal expression of 25-hydroxyvitamin D_3_-1-α-hydroxylase. J. Clin. Endocrinol. Metab..

[10] Chiang K.C., Chen T.C. (2009). Vitamin D for the prevention and treatment of pancreatic cancer. World J. Gastroenterol..

[11] Hewison M. (2012). An update on vitamin D and human immunity. Clini. Endocrinol..

[12] Hewison M., Burke F., Evans K.N., Lammas D.A., Sansom D.M., Liu P., Modlin R.L., Adams J.S. (2007). Extra-renal 25-hydroxyvitamin D_3_-1α-hydroxylase in human health and disease. J. Steroid Biochem. Mol. Biol..

[13] Whitlatch L.W., Young M.V., Schwartz G.G., Flanagan J.N., Burnstein K.L., Lokeshwar B.L., Rich E.S., Holick M.F., Chen T.C. (2002). 25-hydroxyvitamin D-1α-hydroxylase activity is diminished in human prostate cancer cells and is enhanced by gene transfer. J. Steroid Biochem. Mol. Biol..

[14] Schwartz G.G., Eads D., Rao A., Cramer S.D., Willingham M.C., Chen T.C., Jamieson D.P., Wang L., Burnstein K.L., Holick M.F. (2004). Pancreatic cancer cells express 25-hydroxyvitamin d-1α-hydroxylase and their proliferation is inhibited by the prohormone 25-hydroxyvitamin D_3_. Carcinogenesis.

[15] Chiang K.C., Yen C.L., Yeh C.N., Hsu J.T., Chen L.W., Kuo S.F., Wang S.Y., Sun C.C., Kittaka A., Chen T.C. (2015). Hepatocellular carcinoma cells express 25(OH)D-1α-hydroxylase and are able to convert 25(OH)D to 1α,25(OH)(2)D, leading to the 25(OH)D-induced growth inhibition. J. Steroid Biochem. Mol. Biol..

[16] Chiang K.C., Tsui K.H., Chung L.C., Yeh C.N., Chang P.L., Chen W.T., Juang H.H. (2014). Topoisomerase inhibitors modulate gene expression of B-cell translocation gene 2 and prostate specific antigen in prostate carcinoma cells. PLoS ONE.

[17] Bikle D.D., Nemanic M.K., Whitney J.O., Elias P.W. (1986). Neonatal human foreskin keratinocytes produce 1,25-dihydroxyvitamin D_3_. Biochemistry.

[18] Flanagan J.N., Young M.V., Persons K.S., Wang L., Mathieu J.S., Whitlatch L.W., Holick M.F., Chen T.C. (2006). Vitamin D metabolism in human prostate cells: Implications for prostate cancer chemoprevention by vitamin D. Anticancer Res..

[19] McCarthy K., Laban C., Bustin S.A., Ogunkolade W., Khalaf S., Carpenter R., Jenkins P.J. (2009). Expression of 25-hydroxyvitamin D-1-α-hydroxylase, and vitamin D receptor mRNA in normal and malignant breast tissue. Anticancer Res..

[20] Garland C.F., Garland F.C. (1980). Do sunlight and vitamin D reduce the likelihood of colon cancer?. Int. J. Epidemiol..

[21] Schwartz G.G., Hulka B.S. (1990). Is vitamin D deficiency a risk factor for prostate cancer? (hypothesis). Anticancer Res..

[22] Gorham E.D., Garland F.C., Garland C.F. (1990). Sunlight and breast cancer incidence in the USSR. Int. J. Epidemiol..

[23] Garland C.F., Gorham E.D., Mohr S.B., Garland F.C. (2009). Vitamin D for cancer prevention: Global perspective. Ann. Epidemiol..

[24] Brozyna A.A., Jozwicki W., Jochymski C., Slominski A.T. (2015). Decreased expression of CYP27B1 correlates with the increased aggressiveness of ovarian carcinomas. Oncol. Rep..

[25] Tsai M.J., O’Malley B.W. (1994). Molecular mechanisms of action of steroid/thyroid receptor superfamily members. Annu. Rev. Biochem..

[26] Carlberg C., Campbell M.J. (2013). Vitamin D receptor signaling mechanisms: Integrated actions of a well-defined transcription factor. Steroids.

[27] Yang J., Goetz D., Li J.Y., Wang W., Mori K., Setlik D., Du T., Erdjument-Bromage H., Tempst P., Strong R. (2002). An iron delivery pathway mediated by a lipocalin. Mol. Cell.

[28] Candido S., Maestro R., Polesel J., Catania A., Maira F., Signorelli S.S., McCubrey J.A., Libra M. (2014). Roles of neutrophil gelatinase-associated lipocalin (NGAL) in human cancer. Oncotarget.

[29] Bolignano D., Donato V., Lacquaniti A., Fazio M.R., Bono C., Coppolino G., Buemi M. (2010). Neutrophil gelatinase-associated lipocalin (NGAL) in human neoplasias: A new protein enters the scene. Cancer Lett..

[30] Chakraborty S., Kaur S., Guha S., Batra S.K. (2012). The multifaceted roles of neutrophil gelatinase associated lipocalin (NGAL) in inflammation and cancer. Biochim. Biophys. Acta.

[31] Chiang K.C., Yeh C.N., Chen H.Y., Lee J.M., Juang H.H., Chen M.F., Takano M., Kittaka A., Chen T.C. (2011). 19-Nor-2α-(3-hydroxypropyl)-1α,25-dihydroxyvitamin D_3_ (MART-10) is a potent cell growth regulator with enhanced chemotherapeutic potency in liver cancer cells. Steroids.

