# Supplementary material for: 25(OH)D Is Effective to Repress Human Cholangiocarcinoma Cell Growth through the Conversion of 25(OH)D to 1α,25(OH)2D3
Source: Int J Mol Sci. 2016 Aug 12;17(8):1326. doi: 10.3390/ijms17081326 (PMC5000723; doi:10.3390/ijms17081326)
Supplement: Supplementary file 1 [file ijms-17-01326-s001.pdf]

Supplementary Materials: 25(OH)D Is Effective to Repress Human Cholangiocarcinoma Cell Growth through the Conversion of 25(OH)D to 1α,25(OH)$_2$D$_3$

Kun-Chun Chiang, Chun-Nan Yeh, Cheng-Cheng Huang, Ta-Sen Yeh, Jong-Hwei S. Pang, Jun-Te Hsu, Li-Wei Chen, Sheng-Fong Kuo, Atsushi Kittaka, Tai C. Chen and Horng-Heng Juang

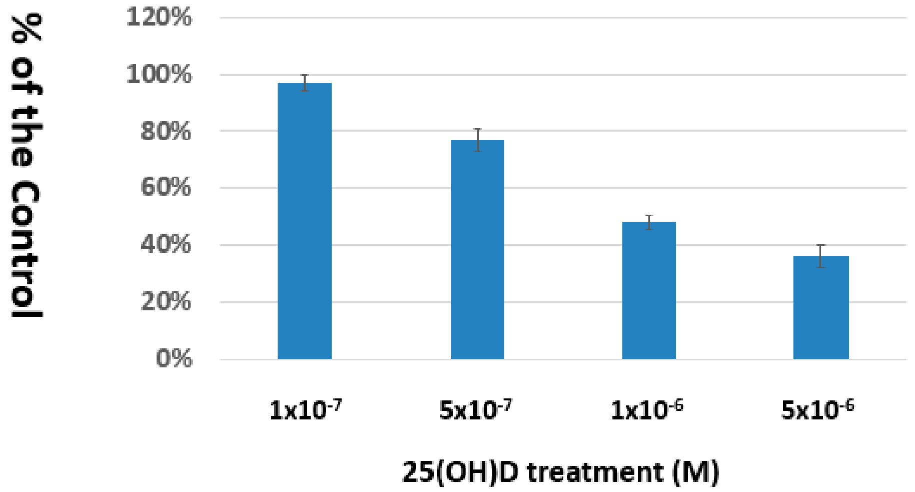

Figure S1. The effect of 25(OH)D on SNU308 cell growth was evaluated by CyQUANT proliferation assay kit. As shown in Figure 1, 1 × 10^{-7}, 5 × 10^{-7}, 1 × 10^{-6}, and 5 × 10^{-6} M 25(OH)D treatments (7 days) suppressed SNU1079 308 cell growth to 97% ± 3%, 77% ± 4%, 48% ± 3%, and 36% ± 4% of the control, respectively.
